# Artificial Neural Networks Coupled with MALDI-TOF MS Serum Fingerprinting To Classify and Diagnose Pathological Pain Subtypes in Preclinical Models

**DOI:** 10.1021/acschemneuro.2c00665

**Published:** 2022-12-30

**Authors:** Meritxell Deulofeu, Eladia M. Peña-Méndez, Petr Vaňhara, Josef Havel, Lukáš Moráň, Lukáš Pečinka, Anna Bagó-Mas, Enrique Verdú, Victoria Salvadó, Pere Boadas-Vaello

**Affiliations:** † Research Group of Clinical Anatomy, Embryology and Neuroscience (NEOMA), Department of Medical Sciences, University of Girona, Girona, Catalonia 17003, Spain; ‡ Department of Chemistry, Faculty of Science, Masaryk University, Kamenice 5/A14, 625 00 Brno, Czech Republic; § Department of Histology and Embryology, Faculty of Medicine, Masaryk University, 62500 Brno, Czech Republic; ∥ Department of Chemistry, Analytical Chemistry Division, Faculty of Sciences, University of La Laguna, 38204 San Cristóbal de La Laguna, Tenerife, Spain; ⊥ International Clinical Research Center, St. Anne’s University Hospital, 656 91 Brno, Czech Republic; # Research Centre for Applied Molecular Oncology (RECAMO), Masaryk Memorial Cancer Institute, 62500 Brno, Czech Republic; ∇ Department of Chemistry, Faculty of Science, University of Girona, 17071 Girona, Catalonia, Spain

**Keywords:** neuropathic pain, fibromyalgia, mass spectrometry, artificial intelligence, MALDI-TOF MS, diagnostics

## Abstract

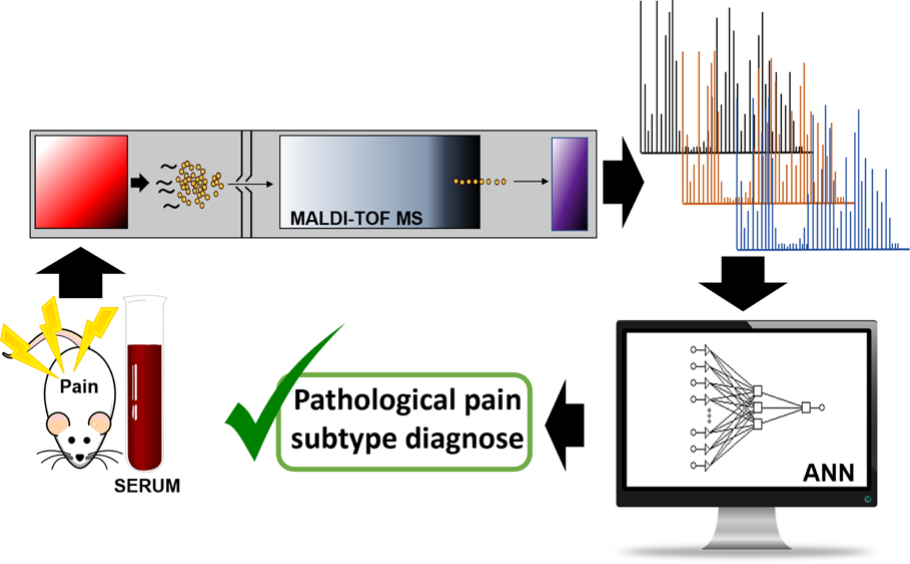

Pathological pain subtypes can be classified as either neuropathic pain, caused by a somatosensory nervous system lesion or disease, or nociplastic pain, which develops without evidence of somatosensory system damage. Since there is no gold standard for the diagnosis of pathological pain subtypes, the proper classification of individual patients is currently an unmet challenge for clinicians. While the determination of specific biomarkers for each condition by current biochemical techniques is a complex task, the use of multimolecular techniques, such as matrix-assisted laser desorption/ionization time-of-flight mass spectrometry (MALDI-TOF MS), combined with artificial intelligence allows specific fingerprints for pathological pain-subtypes to be obtained, which may be useful for diagnosis. We analyzed whether the information provided by the mass spectra of serum samples of four experimental models of neuropathic and nociplastic pain combined with their functional pain outcomes could enable pathological pain subtype classification by artificial neural networks. As a result, a simple and innovative clinical decision support method has been developed that combines MALDI-TOF MS serum spectra and pain evaluation with its subsequent data analysis by artificial neural networks and allows the identification and classification of pathological pain subtypes in experimental models with a high level of specificity.

## Introduction

Pain is a major health concern as it is one of the most common reasons for people to visit primary care settings,^1^ and chronic pain has long been known to be a main source of human suffering and disability.^2−4^ Pathological pain, which is maladaptive rather than protective, is a common complaint that results from the abnormal functioning of the nervous system.^5^ This pain can be classified as either neuropathic pain (NP), which is a disabling condition resulting from a lesion or disease of the somatosensory nervous system, or nociplastic pain, which is caused by altered nociception without evidence of somatosensory system damage.^6^ While stroke, nerve and central nervous system traumas, or neuropathies are examples of NP,^7^ fibromyalgia syndrome (FMS) stands out as a prototypical nociplastic pain condition.

NP and FMS are both clinically diagnosed using a patient’s history—assessed using different scales and questionnaires—and physical examination.^8−10^ In general, pathological pain is characterized by three main sensory symptoms: hyperalgesia (increased pain from a stimulus that normally provokes pain), allodynia (pain due to a stimulus that does not normally provoke pain), and spontaneous pain (pain that does not originate in response to a stimulus).^11,12^ It is not uncommon for both NP and FMS patients to experience similar sensory phenomena, and hyperalgesia and allodynia are commonly seen in patients suffering from both conditions.^8,13^ Hence, pain responses alone are not usually helpful in discriminating between pathological pain subtypes, and considering that other nonreflexive pain responses such as anxiety and depression have also been considered core symptoms of both FMS^9,14^ and NP,^15,16^ the proper classification of individual patients is still an unmet challenge for clinicians.^16^ Furthermore, since there is no gold standard for the diagnosis of pathological pain subtypes, it is not surprising that these conditions remain difficult to treat, and the combination of the lack of suitable tests and efficient treatments leads to pain chronicity^17^ together with associated mood disorders that negatively affect patients’ quality of life. In this context, given the importance of finding a gold standard diagnostic for pathological pain subtypes that may also improve treatment chances, novel diagnostic approaches are needed.

The development and validation of pain biomarkers for diagnostics has become a major issue in pain research.^18^ However, despite much effort having been focused on the discovery of a specific biomarker for each pathological pain condition, no biomarkers for chronic pain have been validated by the Food and Drug Administration in the USA or the European Medicines Agency.^19^ For many decades, most of the studies designed to analyze biomarkers have used a traditional antibody-based immunoassay approach such as immunohistochemistry, enzyme-linked immunosorbent assay, and western Blot.^20,21^ Nevertheless, these approaches have several limitations, including the fact that only known molecules can be studied and there must be a specific antibody against these molecules for detection to be possible.^22,23^ The ability to study multiple biomarkers involving a wide variety of molecules would certainly be advantageous given the complexity of NP and FMS.^24,25^ In this respect, the high speed, sensitivity, selectivity, and versatility of mass spectrometry (MS), a technique widely used in analytical chemistry, offer robust and precise tools for the discovery of potential biomarkers.^26,27^ Among these MS techniques, matrix-assisted laser desorption/ionization time-of-flight (MALDI-TOF) MS is particularly suited to this objective given that it is able to determine several molecules simultaneously even at very low concentrations. Furthermore, MALDI-TOF can cover a wide mass molecular range with a high throughput and can easily be automated to screen a large number of samples.^28−30^ For the diagnosis of pathological pain, we understand the best currently known strategy to be the use of an untargeted fingerprint approach that aims to reveal changes in the general pattern through the identification of a combination of different identities.^23,25,30^ Moreover, new approaches related to nanoparticle-assisted laser desorption/ionization mass spectrometry (LDI MS) allow high-throughput detection of metabolomic fingerprints for disease classification.^31−34^

Although discrimination of disease-specific molecular patterns can be difficult due to the inherent biological complexity of the samples and instrumental variability,^35,36^ it can be solved by applying artificial neural networks (ANNs) to the fingerprint analyses. ANNs are a mathematical representation of human neural architecture and try to reflect the brain’s capacity for learning and generalization. ANNs have the capacity to model nonlinear systems in which the relationship between the variables is highly complex or even unknown without being significantly affected by signal noise. Therefore, ANNs are well suited for pattern recognition and classification and, hence, for clinical diagnosis.^35−38^ In fact, we have previously demonstrated that the use of MALDI-TOF MS to obtain mass profiles of biological samples combined with artificial intelligence tools allows the discrimination of diseased and healthy blood samples in the case of multiple myeloma and COVID-19 diseases.^39,40^ MALDI-TOF MS methods have also been previously applied to determine potential peptide biomarkers of specific pathological pain,^41−44^ and only a few studies have coupled artificial intelligence methods for diagnostics or prediction of pathological pain.^45−50^ However, to our knowledge, there are no studies focused on fingerprint discrimination of different subtypes of pathological pain, which can be used to develop diagnostic tools for health practitioners.

In the light of the above, this study aimed to analyze whether the information provided by the mass spectra of different pathological pain samples could be used to allow ANNs to classify mass spectral profiles of different subtypes of pathological pain and control samples in animal experimental models. Peripheral neuropathic pain (sciatic nerve chronic constriction-injured mice, CCI),^51^ central neuropathic pain (spinal cord-injured mice, SCI)^52,53^ and nociplastic pain conditions (reserpine-induced myalgia mice, RIM6, and intramuscular acid saline solution injected mice, ASI) were the models used in this study.^54^ Furthermore, the capacity of ANNs to discriminate between the different subtypes of pathological pain was assessed in order to evaluate the potential suitability of MALDI-TOF MS and ANN analysis methodology as a clinical decision support tool for the diagnosis and monitoring of pathological pain conditions.

## Results and Discussion

### All Pathological Models Developed Significant Reflexive Pain Responses When Compared with the Corresponding Healthy Control Groups

**Figure 1:**
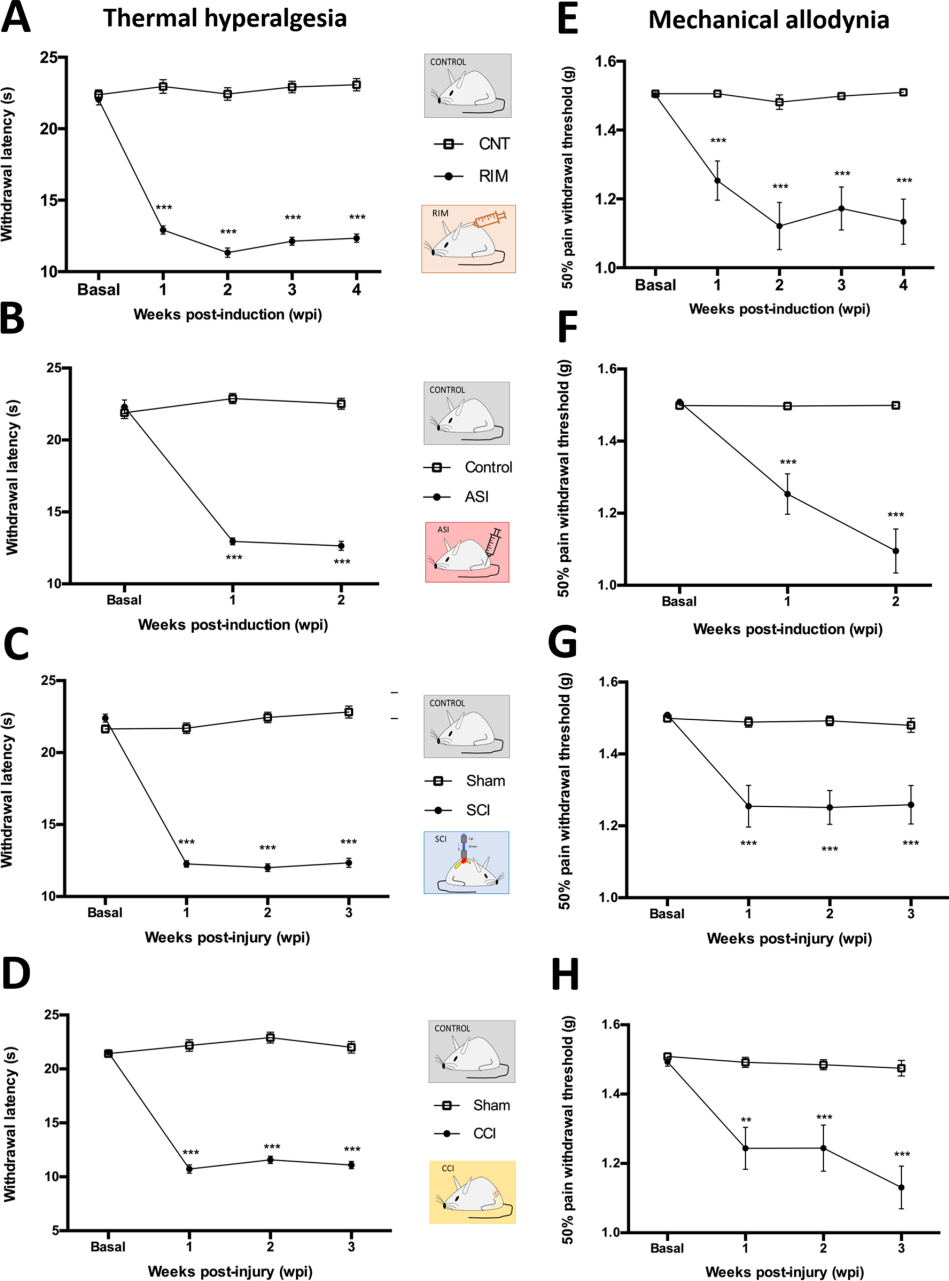
Time-course assessment of thermal hyperalgesia and mechanical allodynia. (A,E) Reserpine-induced myalgia (RIM) model; experimental groups: CNT (*n* = 15) and RIM (*n* = 15). (B,F) Acid-saline induced myalgia (ASI) model; experimental groups: Control (*n* = 15) and ASI (*n* = 15). (C,G) Spinal cord injury (SCI) model; experimental groups: Sham (*n* = 14) and SCI (*n* = 15). (D,H) Chronic constriction injury (CCI) model; experimental groups: Sham (*n* = 15) and CCI (*n* = 15). Each point and vertical line represent the mean ± SEM. ***p < 0.001 significant withdrawal latency decrease vs corresponding control. *p < 0.05; **p < 0.01; ***p < 0.001 significant withdrawal threshold decrease vs corresponding control.

Before any serum analysis, the animals of the four pathological pain models were examined to see whether they had developed reflexive pain responses (thermal hyperalgesia and mechanical allodynia). Thermal hyperalgesia data followed a normal distribution in all models at all the functional assessment time-points (Kolmogorov–Smirnov, all *p* values were above 0.05). Further analysis of variance (ANOVA) tests showed significant differences between the model—RIM, ASI, SCI, and CCI groups—and their respective control groups (*p* < 0.001 in all cases) with the models having significantly decreased paw withdrawal latency to thermal stimulation at all the time-points (Figure 1). With regard to mechanical allodynia, given that the distribution was not normal, nonparametric tests were applied (Kolmogorov–Smirnov, with *p* < 0.05 in all cases). The Mann–Whitney *U* test revealed significant differences (with *p* < 0.01 in all cases) between models and control groups after both the lesion and induction, and these remained significant until the end of the experimental period. Specifically, a significant reduction in the paw withdrawal mechanical thresholds was observed in the models when compared to the corresponding controls (Figure 1). It is worth mentioning that following a protocol for animal welfare supervision,^55^ the general aspect of the animals included in the four animal models was normal, and no changes in coat and skin, vibrissae of nose, nasal secretions, signs of autotomy, weight, or aggressiveness were detected at any time during the experimental period. Hence, it can be concluded that functional data obtained were not related to animal discomfort that might interfere with the functional evaluation.

Taken together, these findings indicate that four pathological pain subtypes were correctly developed, and consequently, specific serum samples from the animal models for peripheral neuropathic pain (CCI), central neuropathic pain (SCI), fibromyalgia-like central nociplastic pain induced (RIM), and fibromyalgia-like peripheral nociplastic pain induced (ASI) were able to be collected to assess the proposed methodology based on the analyses of MALDI-TOF MS fingerprints coupled with ANN. All the models developed reflexive pain responses until the end of the experimental protocol in comparison with their corresponding controls, and these results were consistent with those found in the literature.^51−54,56^

### Mass Spectrum Data Provide Information That May Allow Animals Experiencing Pathological Pain To Be Distinguished from Those That Are Healthy

**Figure 2:**
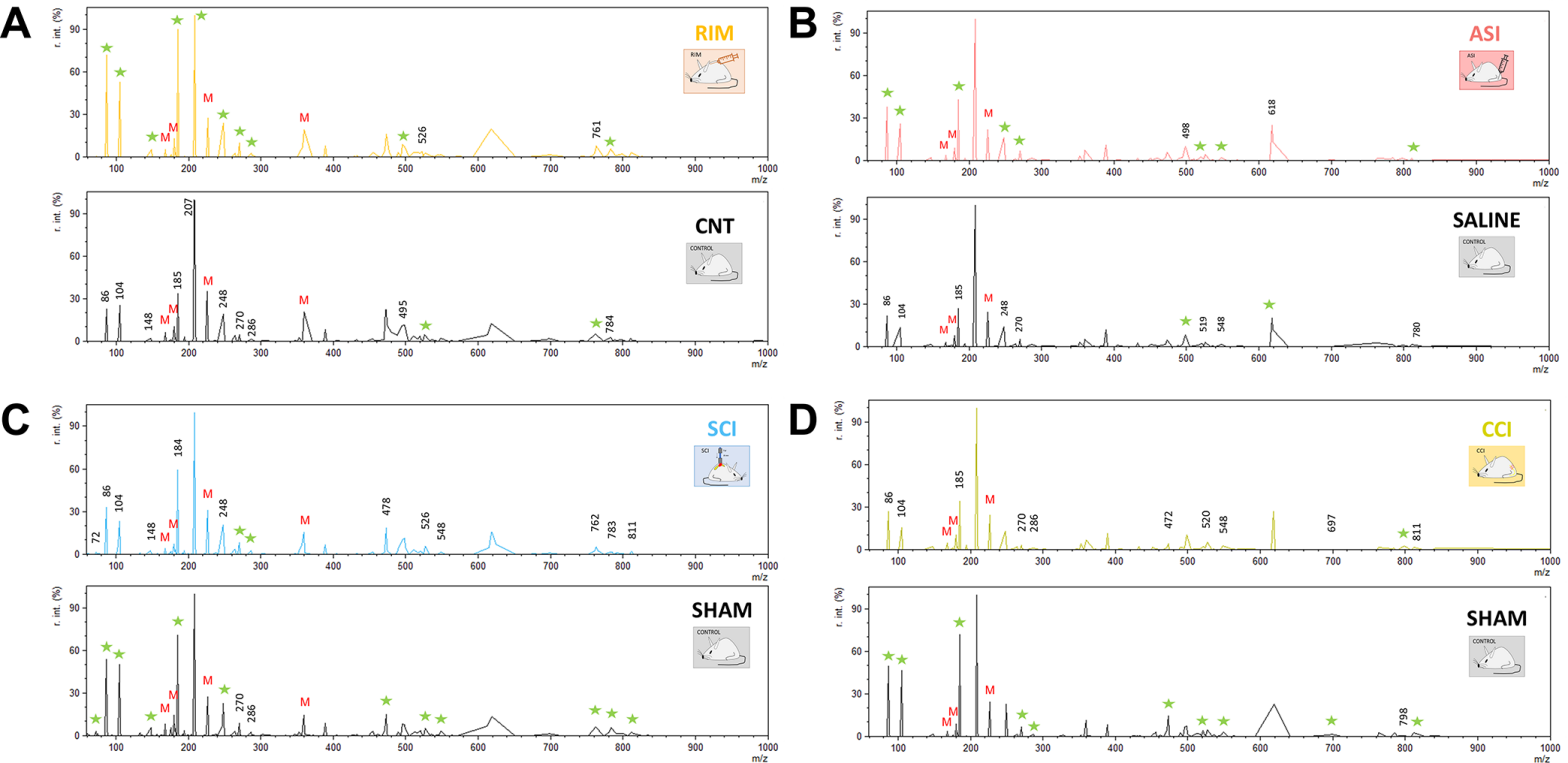
Representative serum mass spectra obtained by MALDI-TOF MS of the different pain animal models. (A) RIM model. (B) ASI model. (C) SCI model. (D) CCI model. Differences between injured—RIM, ASI, SCI, CCI—and non-injured mice can be observed. The stars indicate the group with the highest intensities of some of the signals (in each model). *M* indicated the peaks from the matrix (*M* = matrix).

Collected serum samples from mouse models and controls were analyzed using a MALDI-7090 TOF mass spectrometer in order to obtain mass spectra and determine whether they provide useful information to discriminate between pathological samples and their corresponding controls. The mass spectra fingerprints obtained for all pathological pain models in the positive mode of the injured mice and control mice were similar, and no single peaks seemed to correspond to classmarkers, suggesting that there was no specific biomarker. However, different *m*/*z* regions containing multiple peaks with varying signal intensities between mouse groups were detected (Figure 2).

These results are consistent with current suggestions that the identification of an ideal pain biomarker is still far away.^23^ In fact, establishing a threshold concentration for a specific serum molecule as an endpoint is difficult in these diseases since the molecule concentrations vary significantly depending on the etiology of the particular pain state. In this context, the proposed strategy of measuring several compounds at the same time to identify general patterns,^25^ rather than just single biomolecules, seems to be the most appropriate one to follow. The results obtained indicated that the main differences were in the low mass range (<1000 Da), suggesting potential sample-dependent fingerprints (Figure 2). In this mass range, the peaks are likely to correspond to biomolecules with a low molecular weight or metabolites that could be released from pathophysiological processes of the nervous system that excite nociceptive neurons, and, in turn, cross the blood brain barrier reaching the circulatory system, so allowing their detection in serum samples. These low-molecular-weight molecules may provide important information for the understanding and monitoring of biological processes in disease and other physiological conditions.^57^ Metabolites are the last step in the cascade of omics since these molecules are directly related to the phenotype,^58^ and metabolomics are considered to be the most functional ones.^57^ Changes in low-molecular-weight molecule expression have been reported as possibly being involved in different types of pathological pain. For instance, a decline in antioxidant molecules such as glutathione (307 Da) induces mechanical allodynia and thermal hyperalgesia after CCI,^59−61^ the l-lactate (89 Da) overload resulting from aberrant spinal astrocyte neuron lactate shuttle, has been associated with neuropathic pain maintenance,^62^ and levels of tetrahydrobiopterin (BH4; 241 Da) are dramatically increased in sensory neurons after peripheral nerve damage increasing pain hypersensitivity.^63,64^

Hence, considering that the MALDI TOF MS technique can detect low-molecular-weight biomolecules that may play a pivotal role in pathological pain development and maintenance, a deeper study into low-range peak intensities was performed, finding that this technique generated a large amount of data in which hundreds of *m*/*z* signals were detected in all models. These data were first analyzed using principal component analysis (PCA) to identify potential specific metabolomic patterns underlying the different pathological pain animal models. The samples were plotted using the generated principal components (PCs) on a 3D score to improve visualization and determine whether score plots revealed trends and outliers.^65^

**Figure 3:**
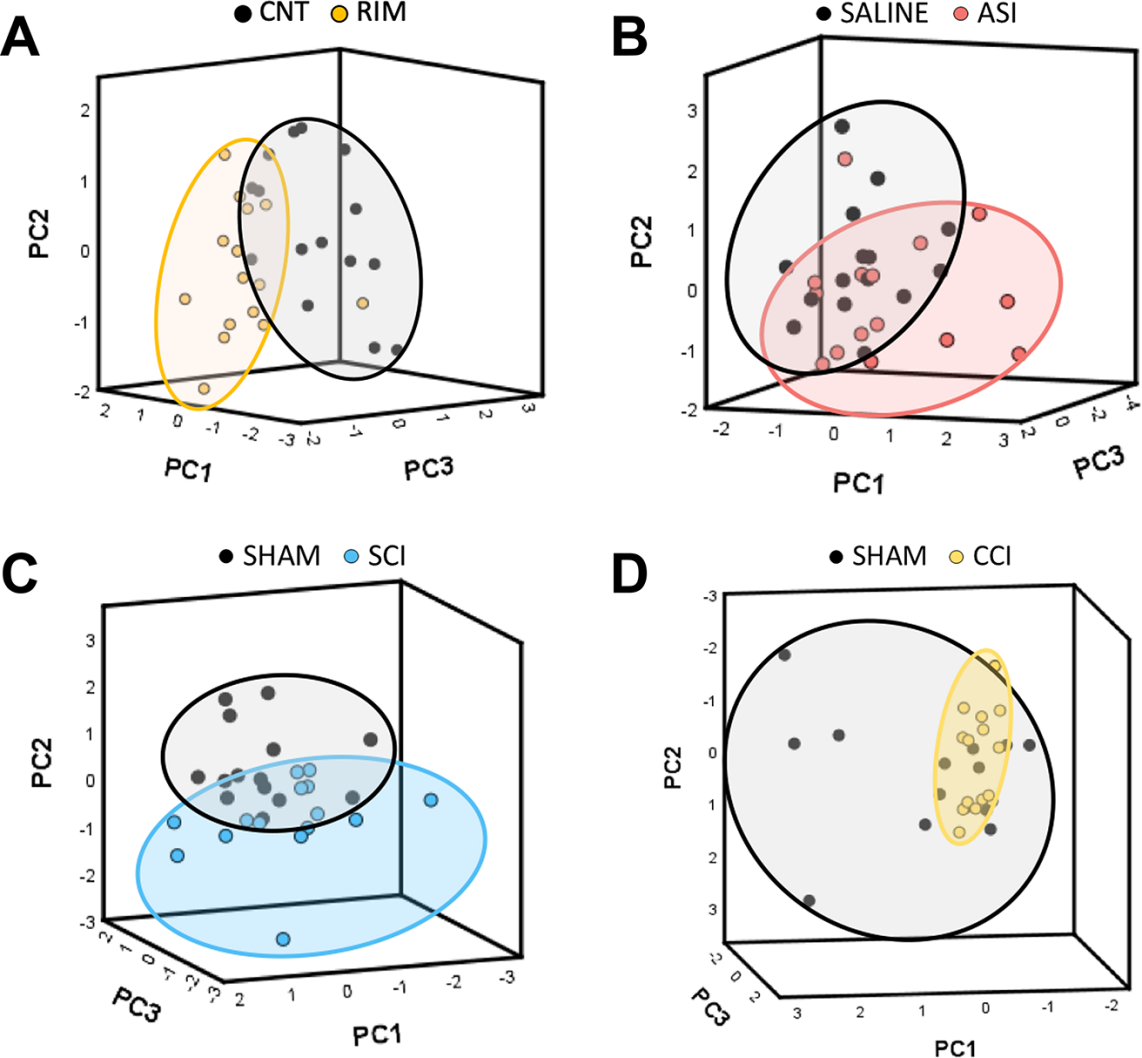
PCA analyses of serum mass spectra obtained with MALDI-TOF of the different pain animal models. (A) RIM model; experimental groups: CNT (*n* = 15) and RIM (*n* = 15). (B) ASI model; experimental groups: saline (*n* = 15) and ASI (*n* = 15). (C) SCI model; experimental groups: Sham (*n* = 14) and SCI (*n* = 15). (D) CCI model; experimental groups: Sham (*n* = 14) and CCI (*n* = 15).

This chemometric study suggested that most pathological pain samples were able to be grouped and distinguished quite clearly from their corresponding controls. Specifically, SCI, RIM, and ASI models were clearly grouped into different clusters although an overlapping area of variable size was observed in all the models (Figure 3A–C). Regarding the CCI model, although CCI samples were grouped in a single cluster, the sham samples were dispersed and did not form a cohesive unit (Figure 3D). Overall, these findings indicate that mass spectrum data provide information that can be used to distinguish animals experiencing pathological pain from those that are not, suggesting also that metabolomic patterns may be present in these mass spectra.

### Serum Fingerprint Analyses by ANNs Discriminate between Samples of Pathological Pain Mouse Models and Their Corresponding Controls

Following on from the previously described results, a new step was performed consisting of combining fingerprints obtained through ANN analyses with the aim of developing a methodology for potential classification and diagnosis. After preparing the database as described in the methodology (Section 2.7 Mass spectrometry data analysis—Artificial neural networks), the characteristic fingerprints were used to optimize the neural network architecture for each model. Since the serum fingerprint of each animal model was different, it is not surprising that the number of relevant *m*/*z* included in their respective datasets was not the same. A total of 53 peaks (*m*/*z*) were included in the CCI-model database while 63, 43, and 73 were included in the SCI-, ASI-, and RIM-model databases, respectively (Figure S1). All the calculations were based on feedforward neural networks operating in a supervised learning mode using back propagation algorithms. Model overfitting, which is one of the main problems in ANN analysis,^37^ was not detected in any of the analyses performed after more than 50,000 training cycles (epochs).

**Figure 4:**
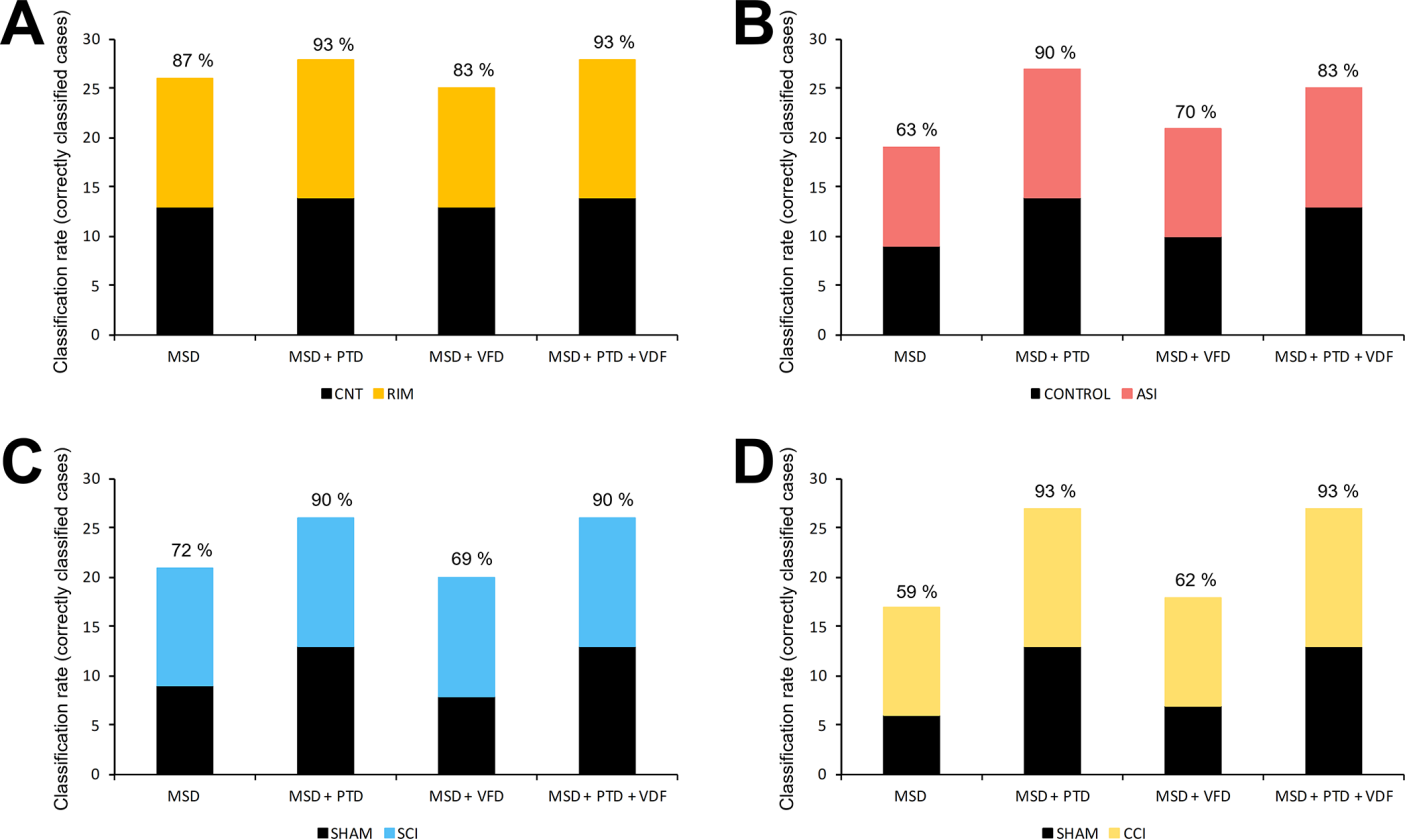
Summary of the ANN classification output. Results of the ANN classification output using the databases built for the different models: (A) RIM model; (B) ASI model; (C) SCI model; and (D) CCI model. Only the correct samples are represented in the plot, and the success percentage is given at the top of each column. The vertical axis indicates the information included from the different database analyzed. MSD = mass spectrum data; PTD = plantar test data; VFD = von Frey test data.

The capacity for prediction and generalization of the ANN models must be verified to confirm that new samples can be classified reliably. Ideally, the model would be validated using completely new data, but given the difficulty in obtaining a secondary independent database when dealing with clinical samples, a cross validation method was used. This method allows the evaluation of the robustness of the model by dividing the samples present in the database into a training set, which is used to build the model during the training phase, and a verification set, which is used as new data to assess the model performance.^57,66^ Thus, in the verification phase of our study, the leave-one-out validation method was performed to confirm the classification model’s ability to predict the output of the excluded sample.^57^ Then, based on the samples that were correctly classified for each pathological pain animal model, a classification output success—expressed as a percentage—was calculated (Figure 4). The RIM fibromyalgia-like model resulted in 87% of correct predictions (Figure 4A). In other words, only two samples from each group were not classified correctly, whereas 26 samples were assigned to their corresponding groups. Regarding the ASI fibromyalgia-like model, 63% of correct predictions were obtained in cross-validation (Figure 4B), since nine controls and 10 ASI mice samples were classified into their corresponding groups. In the SCI central neuropathic pain models, 82% were classified correctly (Figure 4C), whereas in the group of CCI peripheral neuropathic pain and their respective control mice, 59% of correct predictions were obtained (Figure 4D).

Taken together, these results suggest that meaningful information from the mass spectra may be used as inputs for ANN analyses to discriminate between serum samples of pathological models and healthy mice. However, to consider this methodology as a reliable diagnostic tool, some improvements are required to classify results more precisely, especially in the case of peripheral insult-induced pathological pain (CCI and ASI). To this end, outputs from functional analyses were used in addition to fingerprint data.

### Mass Spectra Fingerprints in Combination with Functional Data Improve the Capacity of ANN Models To Successfully Classify Both Pathological Pain and Healthy Samples

It has been shown that different types of input data can be processed together using the ANN to produce a significant output.^35^ In an attempt to improve the percentage of successfully classified samples, the functional data (thermal hyperalgesia and mechanical allodynia outputs) were added to the ANN models. These two variables were first added individually to the different datasets containing the mass spectra data and then both reflexive pain responses variables were included together at the same time.

When the databases containing the mass spectra data and the functional data were analyzed by PCA, few differences were observed in the 3D score plots (Figure S2). On the other hand, remarkable differences were obtained when they were analyzed by the ANN. When thermal hyperalgesia data were included in all the datasets analyzed in the present work, the percentage of successfully classified samples improved in all cases, never falling below 90% (Figure 4). These results were in line with the differences observed in the plantar test between the pain models and their respective controls that were evident in all cases at the end of the experimental protocol (Figure 1). However, when the information of mechanical allodynia was added to the ANN model, the results were not as good as before. In most cases, the percentage of correctly classified samples was lower than when only the information present in the mass spectrum was included in the database (Figure 4). In the particular case of the ASI pain animal model, the percentage did increase but the classification rate was not as high as when the plantar test was added. The counterproductive effect of mechanical allodynia outputs in the ANN model could be explained by the low variability observed in the von Frey data in which results ranged only from 0.8 to 1.51 g. Furthermore, the variance (*s*^2^) was lower than 1 in comparison to the higher variance values that were obtained in the thermal hyperalgesia variable. Finally, when data of both functional variables were included in the datasets, a higher percentage of correctly classified samples (Figure 4) were observed, which in most cases was equal to the results that were obtained when only thermal hyperalgesia data was included. The latter may confirm the low relevance of von Frey data for the model.

Hence, all these results show that mass spectra fingerprints in combination with functional data improve the classification capacity of ANN models. Overall, the results reported so far indicated that by analyzing serum samples using MALDI TOF MS and applying PCA and ANN analyses to the resulting mass spectrum information, CCI, RIM, ASI, and SCI pathological pain samples can be discriminated from their respective controls. Once this milestone had been achieved, the study progressed to the development of a new ANN model that would be able to discriminate between the different pathological pain samples, instead of just between models and control samples, and, hence, potentially provide a diagnostic tool for the identification of pathological pain subtypes. To this end, the serum mass spectra obtained for the four pathological groups included in the study—CCI, SCI, ASI, and RIM groups—were compared and used to develop the new methodology.

### Combination of MALDI-TOF MS and ANNs Is a Suitable Methodology To Discriminate between Pathological Pain Subtypes and May Be a Suitable Clinical Decision Support Tool for the Diagnosis and Monitoring of These Health Conditions

**Figure 5:**
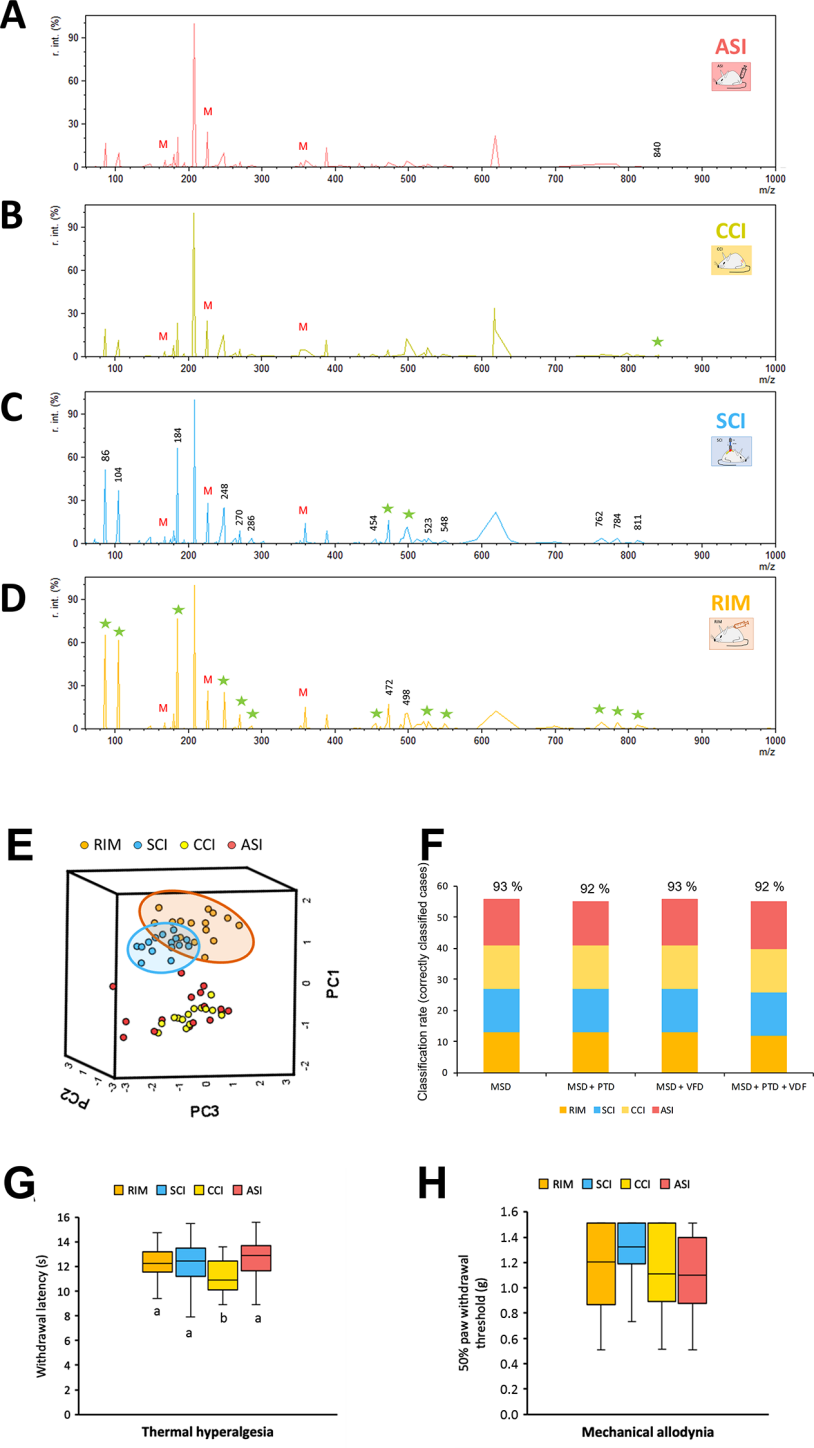
Serum mass spectra and the comparison of all pathological pain experimental models. (A–D) Differences in the serum spectra can be observed between groups. The ASI and CCI mice have with the highest intensity signals which are indicated with stars. (E) Score plot obtained after PCA analysis of the database containing mass spectrum data. (F) Results of the ANN classification output using the different databases built for the comparison of the four pathological groups. Only correctly classified samples are represented in the plot and the success percentage is included at the top of each column. The “*x*” axis shows the different included information in the analyzed database. MSD = mass spectrum data; PTD = plantar test data; VFD = von Frey test data. (G) Comparison of thermal hyperalgesia; data shown as the median ± IQR (interquartile range) for each group; a,b: groups not sharing a letter are significantly different, *p* < 0.05, by Duncan’s test. (H) Comparison of mechanical allodynia data. No statistical differences were found. Experimental groups: RIM (*n* = 15), SCI (*n* = 15), CCI (*n* = 15), and ASI (*n* = 15).

When serum samples were analyzed and the resulting mass spectra were compared, no single peaks that might correspond to specific classes were found (Figure 5A–D). However, similarly to the previous experiments, several peaks of varying intensities within the four models were observed, suggesting that the metabolomic patterns of pathological pain subtypes could be different and specific for each condition. Hence, these results may give further support to the hypothesis that the analysis of a group of molecules could be a better approach than the identification of single biomarkers for the study of pathological pain. Specifically, a total of 74 *m*/*z* signals corresponding to relevant peaks were selected for inclusion in the dataset (Figure S3). Then, when the mass spectra data were analyzed by PCA and samples were represented on a 3D score plot, only SCI and RIM samples were grouped together in the case of the first three PCs, forming two different clusters, where ASI and CCI samples were mixed (Figure 5E). Despite the latter finding, the resulting new ANN generated model based on mass spectra outputs was able to discriminate between pathological pain subtype samples with high specificity (93%) (Figure 5F), as only four samples (2 RIM, 1 SCI, and 1 CCI) were classified as unknown.

Although these results were promising, it was decided to add the reflexive pain responses outputs also since this additional information had made it possible to improve the discrimination between pathological pain models and their controls in the previous experiments. Before ANN analysis, thermal hyperalgesia and mechanical allodynia were compared between the different pathological groups to determine whether differences between the experimental group were present. The statistical analysis of thermal hyperalgesia data showed significant group differences. However, no significant differences were shown between most groups (*p* > 0.05 in all cases) and only CCI mice showed significantly decreased paw withdrawal latency to thermal stimulation in comparison with RIM, SCI, and ASI groups (*p* < 0.05 in all cases) (Figure 5G).

On the other hand, mechanical allodynia data analysis showed a lack of significant differences in the paw withdrawal mechanical threshold between groups (Figure 5H). These reflexive pain variables, which indicated similar reflexive pain responses in all groups, were included separately in the database. However, in contrast to the previous studies, no differences were observed in the corresponding score plots after the PCA analyses (Figure S2). Furthermore, when the thermal hyperalgesia data were subsequently added to generate the ANN model, 92% of the samples were correctly classified and 93% of correct classifications was reached when mechanical allodynia data were included (Figure 5F). Therefore, the addition of one or both reflexive pain responses variables had little effect on the sample classification in the newly generated ANN model.

In summary, all these findings confirm that MALDI TOF MS serum analysis and its subsequent data analysis by ANNs is a suitable methodology to discriminate between subtypes of pathological pain, even without including reflexive pain response outputs. This is really interesting since these results could be seen as mimicking what happens in the case of humans given that hyperalgesia and mechanical allodynia are two of the main clinical manifestations of both NP^11^ and FMS patients^13^ and consequently they would not be clinically discriminative on their own. In addition, FMS usually present neuropathic pain features^67^ and other phenotypic similarities that have been detected in patients of both aetiologies^12,67^ causing patients to choose very similar descriptors to define their sensory perceptions. Thus, while using pathological pain response data to differentiate between subtypes conditions would not be helpful, the new methodology generated in this study has the capability of discriminating between pathological pain subtypes, so minimizing the need for reflexive pain response variables.

## Conclusions

An innovative, simple, and fast method for the detection and classification of pathological pain subtypes in experimental models using serum mass spectra has been developed. Moreover, the analysis of pain responses outcomes and MALDI-TOF MS serum spectra in combination with ANNs provides a methodology for the detection of pathological pain subtypes and the identification of their origin through their fingerprints without the need for the identification of single biomarkers. These findings may usefully be translated into clinical practice in using the MALDI-TOF MS ANN methodology as a decision support tool for the diagnosis and monitoring of pathological pain subtypes. Finally, but not least importantly, the developed methodology may also be used for the detection of molecules involved in the generation and persistence of pathological pain that could become potential therapeutic targets.

## Methods

### Drugs, Reagents, and Solutions

Reserpine (metil-11,17α-dimetoxi-18β-((3,4,5-trimetobenzoilo)oxy)-3β,20α-yohimban-16β-carboxylate) (R0875, Sigma Aldrich, USA) was dissolved in glacial acetic acid (A6283; Sigma-Aldrich, USA) and diluted to a final concentration of 0.25% acetic acid with distilled water.^54^ Sterile saline solution was mixed with the acid (A6283; Sigma-Aldrich, USA) until pH = 4 was reached.^54^ Sinapinic acid (3,5-Dimethoxy-4-hydroxycinnamic acid) (D7927, Sigma-Aldrich, Germany) was used as the matrix for MALDI-TOF MS analysis. Acetonitrile (ACN) (271004, Merck, Germany) and trifluoroacetic acid (TFA) (T6508, Sigma-Aldrich, Germany) were used for matrix preparation. Micro-90 concentrated cleaning solution (Z281506, Sigma-Aldrich, Germany) was used for MALDI-TOF MS target cleaning between different analyses.^38^ Red phosphorus (04004H, Riedel de Haën, Germany) was used for MALDI-TOF MS calibration.^68^

### Animals

Eight-week-old female ICR-CD1 mice (20–30 g) were obtained from Janvier Laboratories (France). The number of mice used was kept to a minimum, working with experimental groups each consisting of 14–15 mice. The animal sample size needed for functional evaluation was calculated using GRANMO (Version 7.12 April 2012) and the University of Boston spreadsheet (Sample Size Calculations (IACUC); Boston University) within the ethical limits set by the Animal Ethics Committee. Mice were housed in standard plexiglass cages (28 × 28 × 15 cm) with free access to food and water, with a 12:12 h light/dark cycle, a temperature of 21 ± 1 °C, and 40–60% of humidity. Cages were changed twice weekly. All mice were allowed to acclimatize for at least 1 h to the facility rooms before any functional or surgical procedures, which were all conducted during the light cycle. Sentinel mice were routinely tested for pathogens, and facilities remained pathogen-free during the whole experimental period.

All experimental procedures and animal husbandry were conducted following the ARRIVE 2.0 guidelines and according to the ethical principles of the I.A.S.P. for the evaluation of pain in conscious animals,^69^ which are contained in the European Parliament and Council Directive of 22 September 2010 (2010/63/EU). The study protocol was also approved by the Animal Ethics Committee of the University of Barcelona and the *Generalitat de Catalunya*, Government of Catalonia (DAAM numbers 8884 and 8887). All efforts were made to minimize animal suffering and to keep the number of animals to a minimum to demonstrate consistent effects for the procedures.

### Experimental Design and Animal Models

To analyze the different subtypes of pathological pain, four independent studies were conducted with experimental models of neuropathic and nociplastic pain. Peripheral neuropathic pain was induced in mice by the ligation of the sciatic nerve performed in accordance with procedures described elsewhere.^51,70^ Briefly, animals were first anesthetized with sodium pentobarbital (50 mg/kg, i.p.), and an incision was made in the right thigh exposing the sciatic nerve. Two ligatures 1 mm apart were then made around the exposed nerve causing a chronic constriction injury (CCI; CCI group). Finally, the incision was closed using 5–0 interrupted nylon sutures. A sham group in which the surgery was performed, the sciatic nerve was exposed but no further manipulation was made was also established for this first experiment. A second set of mice was used to analyze central neuropathic pain induced by mild SCI. After anesthetizing the animals, spinal cord contusion was performed with a device to drop weights following a procedure explained elsewhere^52,53^ that allows central neuropathic pain to be induced without leading to animal paralysis. After a dorsal laminectomy, T8–T9 thoracic spinal cord segments were exposed, and 2 g of weight was dropped from a height of 25 mm onto a metallic stage located over the exposed spinal cord (SCI group). Following this procedure, the wound was closed, and the animals were kept in a warm environment until full recovery. Animals also received 0.5 mL of saline solution to restore an eventual blood volume deficit. In the corresponding sham group, the spinal cord was exposed but not contusioned. Regarding nociplastic pain, two different fibromyalgia-like pain animal models were used. For the RIM model, the RIM6 model was induced.^54^ Briefly, reserpine (Sigma-Aldrich; St. Louis, MO, USA) dissolved in acetic acid and diluted to a final concentration of 0.5% acetic acid with saline solution was administered subcutaneously (0.25 mg/kg) on days 0, 1, 2, 9, 16, and 23 (RIM group). The corresponding controls (CNT) received the reserpine dilution vehicle subcutaneously at the same time-points. The second model of nociplastic pain was the acid saline-induced (ASI) myalgia. In this case, a volume of 10 μL of acidic saline solution at pH 4 was administered intramuscularly using a Hamilton syringe into the right gastrocnemius muscle (ASI group) at days 0 and 5.^54^ Sterile saline solution was administered under the same conditions in the corresponding control mice group.

### Functional Evaluation

Functional tests were performed before starting the experimental protocol and once per week until the end of the experimental period of each pathological pain experimental model. Regarding reflexive pain response assessments, the Hargreaves and von Frey tests were performed to evaluate thermal hyperalgesia and mechanical allodynia, respectively. For thermal hyperalgesia evaluation, a plantar algesimeter (#37370; Ugo Basile, Comerio, Italy) was used in accordance with the Hargreaves method.^53,71^ Mice were placed into a plastic box with an elevated glass floor and allowed to acclimate for 1 hour. The light of a projection lamp (100 W) was then focused directly onto the plantar surface of the hind paw. The time to withdrawal of the heated paw (withdrawal latency) was measured through a time-meter coupled with infrared detectors directed at the plantar surface. A cut-off time of 25 s was imposed to avoid skin damage. The result was established as the mean of at least three trials separated by 5-min resting periods. In the CCI model, the withdrawal latency of the injured paw was measured, whereas in the other models both paws were analyzed as the lesions could result in bilateral injuries. Mechanical allodynia was assessed using the hind paw withdrawal response to von Frey filament stimulation.^56,72^ Mice were placed on different plastic tubes on a framed metal mesh floor and allowed to acclimate for 1 hour. Von Frey monofilaments (bending force range from 0.04 to 2 g) were then applied onto the plantar surface of the hind paws, and thresholds were measured using the up-down method paradigm. Initially, the 0.4 g filament was used but the strength of the next filament was then decreased or increased depending on whether the mouse responded. The procedure was stopped four measurements after the first response of the animal. A clear paw withdrawal, shaking or licking was considered a positive response. Each filament was applied for 2 s at intervals of about 5–10 s between each stimulation. As in the previous test, both paws were measured in all models except in the CCI model, where only the injured leg was measured. The 50% paw withdrawal threshold was calculated using the Dixon formula: 50% paw withdrawal threshold (*g*) = ((10^(Xf+κδ)^/10,000)), where Xf is the value (in logarithmic units) of the final von Frey filament used, κ is a fixed tabular value for the pattern of positive/negative responses, and δ is the mean difference (in log units) between stimuli.

All functional analyses were blinded using a numerical code for each mouse. Functional data were analyzed using repeated measurements. MANOVA (Wilks’ criterion) and ANOVA or the Friedman statistical test for nonparametric repeated measures followed by the Mann–Whitney *U* test were used when the data did not follow a normal distribution. The SPSS 25.0 for Windows statistical package was used for all analyses and significance was set at 0.05.

### Sample Collection and Preparation for MS Analysis

At the end of the experimental protocol, all the animals were anesthetized with sodium pentobarbital (90 mg/kg; i.p.), and blood was collected through the insertion of an intracardiac needle. This was then centrifuged for 15 min at 4000 rpm to obtain serum, which was immediately frozen in dry ice and stored at −80 °C until analysis by MALDI-TOF MS. For MS analysis, the serum samples (maximum 10 μL) were first diluted 10 times with double distilled water (dd-H_2_O) and mixed at a 1:1 ratio with a solution of sinapinic acid (SA) containing 20 mg SA /mL in 60%:40% (v/v) acetonitrile (ACN): dd-H_2_O with 0.3% trifluoroacetic acid (TFA) to increase the ionization. 1 μL of the mixture was then placed on a purified stainless-steel target plate in triplicate.^38^ The protocol for sample preparation was previously optimized by analyzing serum samples using different matrices and dilutions.

### Acquisition of Mass Spectra

Mass spectra were acquired using a MALDI-7090 TOF mass spectrometer from Kratos Analytical Ltd. (Manchester, UK) equipped with a nitrogen laser operating at 355 nm, delayed extraction, and a microchannel plate detector. The laser energy was expressed in arbitrary units (a.u.) and set at 140 a.u. The laser fluence was ≈10 mJ/mm^2^/pulse, the accelerating voltage was set at 20 kV, and laser repetition at 5 Hz with a pulse time width of 3 ns. All measurements were carried out in positive linear mode and the mass range, which was from 0 to 10,000 Da, was analyzed. The automatic mode was set to record all mass spectra using a regular raster, and the spectra were registered as the relative ion signal to the mass-to-charge (*m*/*z*) value. The spectra were normalized, establishing maximum peak intensity equal to 100%. Moreover, matrix samples were used as blanks and were analyzed to differentiate the matrix peaks from those of the samples. Solutions software from Kratos Analytical Ltd. was used to evaluate and export the mass spectra.

### Mass Spectrometry Data Analysis: Artificial Neural Networks

After mass spectra exportation, data were preprocessed using the R Studio software. The preprocessing of the data consisted of the removal of the background, normalization of signal intensity, smoothing and baseline subtracting using Savitzky–Golay and Loess method, respectively, and spectra alignment. The goal of this preprocessing step was to reduce the variance within databases.^73^ An individual database was built to analyze the mass spectra obtained from each pain animal model using R Studio software. Thus, four different databases were constructed for the four different pain models. A fifth database was built to compare the spectral fingerprints of these four models. The resulting files, which contained all the information of the mass spectra including nonrelevant information, were revised and cleaned before starting the statistical analyses. To this end, the variance (*s*^2^) of the mean intensity of the different peaks was calculated, and only those with a *s*^2^ > 1 were included in the final database. Z-scaling was then applied to each of the datasets, and PCA was performed to analyze databases using the SPSS 25.0 statistical package. The main *m*/*z* variables for each database, as well as the functional data for some experiments, were selected to construct PCAs using TRAJAN 3 software (Trajan Software Ltd., Trajan House, Lincs, UK), and these were used to classify the models. The multilayer perceptron network was the architecture used for all the experiments in the study. Briefly, this type of ANN consists of several artificial neurons or nodes organized in one input layer, one or more hidden layers, and one output layer. First, the best architecture for each pathological pain subtype model was optimized before each analysis. Once the parameters of the learning model were fixed, the number of nodes in the hidden layer were chosen to minimize the root mean squared error. In all the experiments included in this work, an architecture with three nodes in the hidden layer was used. The inputs of all the networks used were the intensities of the selected *m*/*z* signals of the animals included in the database. The number of inputs were specific for each model. The network, and therefore the classification model, was trained using the back-propagation algorithm with a maximum number of iterations of 50,000 and a classification confidence level of 0.05. After the training phase, each model was verified using the leave-one-out cross-validation method to test the prediction capacity of the model in classifying single samples that were excluded from the training data set. Cases that were not identified by ANN and, hence, whose output was unknown, were classified as erroneous predictions.

## Supporting Information Available

Intensities of the most relevant peaks found in mass spectra of serum samples obtained with the MALDI-TOF of (A) CCI and sham, (B) SCI and sham, (C) ASI and saline, and (D) RIM and CNT; data shown as the median of each group ± IQR, a significant difference in only one peak (**p* < 0.05); analyses of the serum mass spectrum data obtained with MALDI-TOF and pain response data of the different pain animal models; score plots on which samples are represented in terms of the first three principal components; graphs obtained after the PCA calculations using the different databases containing the mass spectrum data and (A) thermal hyperalgesia data, (B) mechanical allodynia data, or (C) both variables; intensities of the most relevant peaks found in mass spectra from injured mice serum samples obtained with the MALDI-7090 TOF; data shown as the median of each group ± IQR, a significant difference found in only one peak; and a–c: groups not sharing a letter in the same peak are significantly different, *p* < 0.05, by Duncan’s or Kruskal–Wallis test (PDF)
