# Supplementary material for: Artificial Neural Networks Coupled with MALDI-TOF MS Serum Fingerprinting To Classify and Diagnose Pathological Pain Subtypes in Preclinical Models
Source: ACS Chem Neurosci. 2022 Dec 30;14(2):300–11. doi: 10.1021/acschemneuro.2c00665 (PMC9853500; doi:10.1021/acschemneuro.2c00665)

Supporting Information for:

Artificial neural networks coupled with MALDI-TOF MS serum fingerprinting to classify and diagnose pathological pain subtypes in preclinical models

Meritxell Deulofeu, [†,‡,§] *Eladia M. Peña-Méndez,* [||] *Petr Vaňhara,* [§ #] *Josef Havel* [‡,#] *Lukáš Moráň,* [§,ǂ] *Lukáš Pečinka,* [‡,#] *Anna Bagó-Mas,* [†] *Enrique Verdú,* [†] *Victoria Salvadó,* [*,$] *and Pere Boadas-Vaello* [*,†]

[†]Research Group of Clinical Anatomy, Embryology and Neuroscience (NEOMA), Department of Medical Sciences, University of Girona, Girona, Catalonia, Spain

[‡]Department of Chemistry, Faculty of Science, Masaryk University, Kamenice 5/A14, 625 00 Brno, Czech Republic

[§]Department of Histology and Embryology, Faculty of Medicine, Masaryk University, 62500 Brno, Czech Republic

[||]Department of Chemistry, Analytical Chemistry Division, Faculty of Sciences, University of La Laguna, 38204 San Cristóbal de La Laguna, Tenerife, Spain

[#]International Clinical Research Center, St. Anne's University Hospital, 656 91 Brno, Czech Republic

[ǂ]Research Centre for Applied Molecular Oncology (RECAMO), Masaryk Memorial Cancer Institute, Brno, Czech Republic.

[$]Department of Chemistry, Faculty of Science, University of Girona, 17071 Girona, Catalonia, Spain

***Corresponding authors:** Dr. Pere Boadas-Vaello and Dr. Victoria Salvadó.
pere.boadas@udg.edu and victoria.salvado@udg.edu

SUPPORTING FIGURES

Fig. S1 shows Intensities of the most relevant peaks found in mass spectra of serum samples obtained with the MALDI-TOF of (**A**) CCI and sham, (**B**) SCI and sham, (**C**) ASI and saline and (**D**) RIM and CNT. Data shown as the median of each group ± IQR. A significant difference was found in only one peak. (* $p<0.05$).

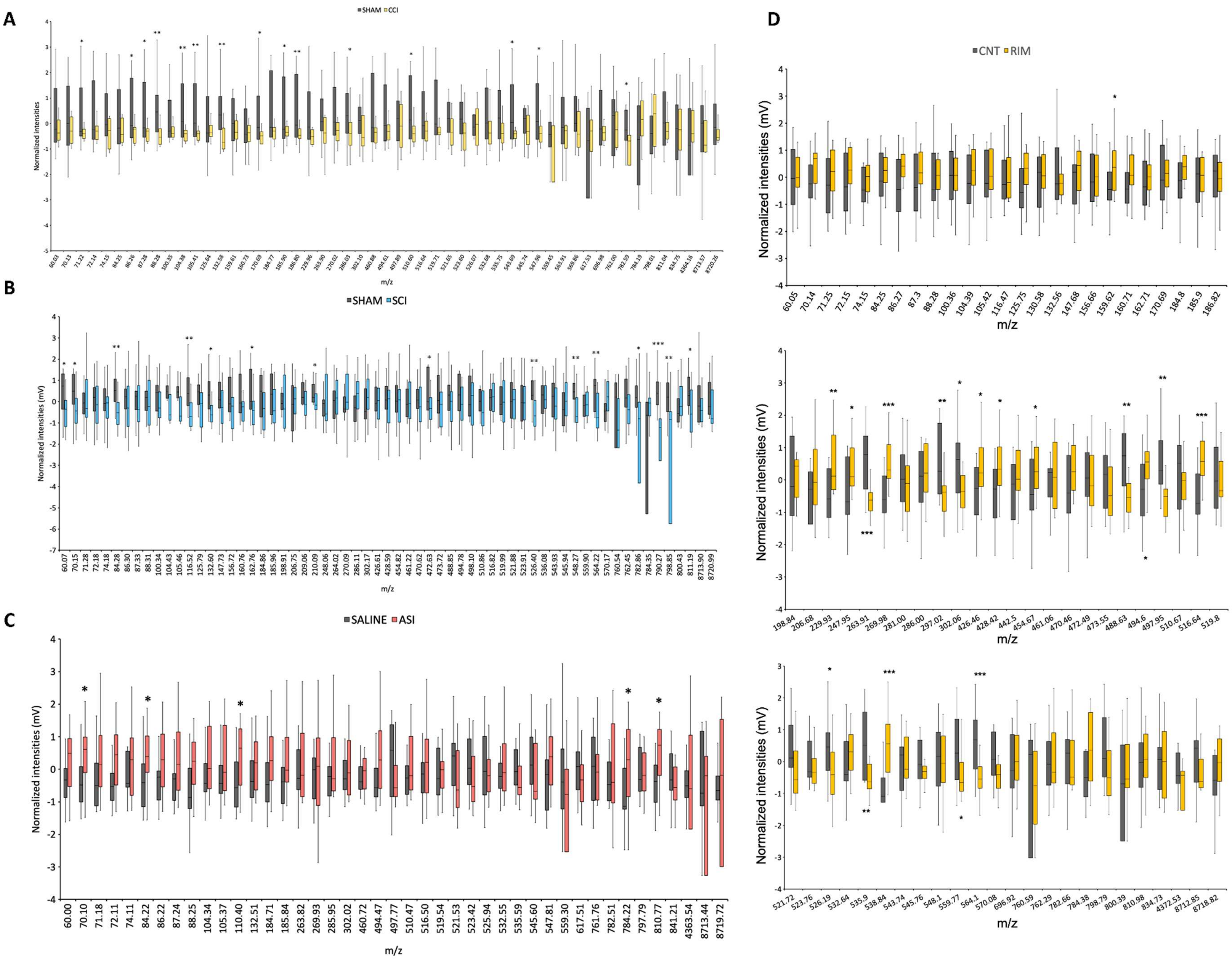
A
SHAM CCI
Normalized intensities
m/z
B
SHAM SCI
Normalized intensities (mV)
m/z
C
SALINE ASI
Normalized intensities (mV)
60.00 70.10 71.18 72.11 74.11 84.22 86.22 87.24 88.25 104.34 105.37 110.40 132.51 184.71 185.84 263.82 269.93 285.95 302.02 460.72 494.47 497.77 510.47 516.50 519.54 521.53 523.42 525.94 532.55 535.59 545.60 547.81 559.30 617.51 761.76 782.51 784.22 797.79 810.77 841.21 4363.54 8713.44 8719.72
m/z
D
CNT RIM
Normalized intensities (mV)
60.05 70.14 71.25 72.15 74.15 84.25 86.27 87.3 88.28 100.36 104.39 105.42 116.47 125.75 130.58 132.56 147.68 156.66 159.62 160.71 162.71 170.69 184.8 185.9 186.82
m/z
Normalized intensities (mV)
198.84 206.68 229.93 247.95 263.91 269.98 281.00 286.00 297.02 302.06 426.46 428.42 442.5 454.67 461.06 470.46 472.49 473.55 488.63 494.6 497.95 510.67 516.64 519.8
m/z
Normalized intensities (mV)
521.72 523.76 526.19 532.64 535.9 538.84 543.74 545.76 548.1 559.77 564.1 570.08 696.92 760.59 762.29 782.66 784.38 798.79 800.39 810.98 834.73 4372.53 8712.85 8718.82
m/z

Fig. S2 shows the analyses of the serum mass spectrum data obtained with MALDI-TOF and pain response data of the different pain animal models. Score plots on which samples are represented in terms of the first three principal components. Graphs were obtained after the PCA calculations using the different databases containing the mass spectrum data and (**A**) thermal hyperalgesia data, (**B**) mechanical allodynia data or (**C**) both variables.

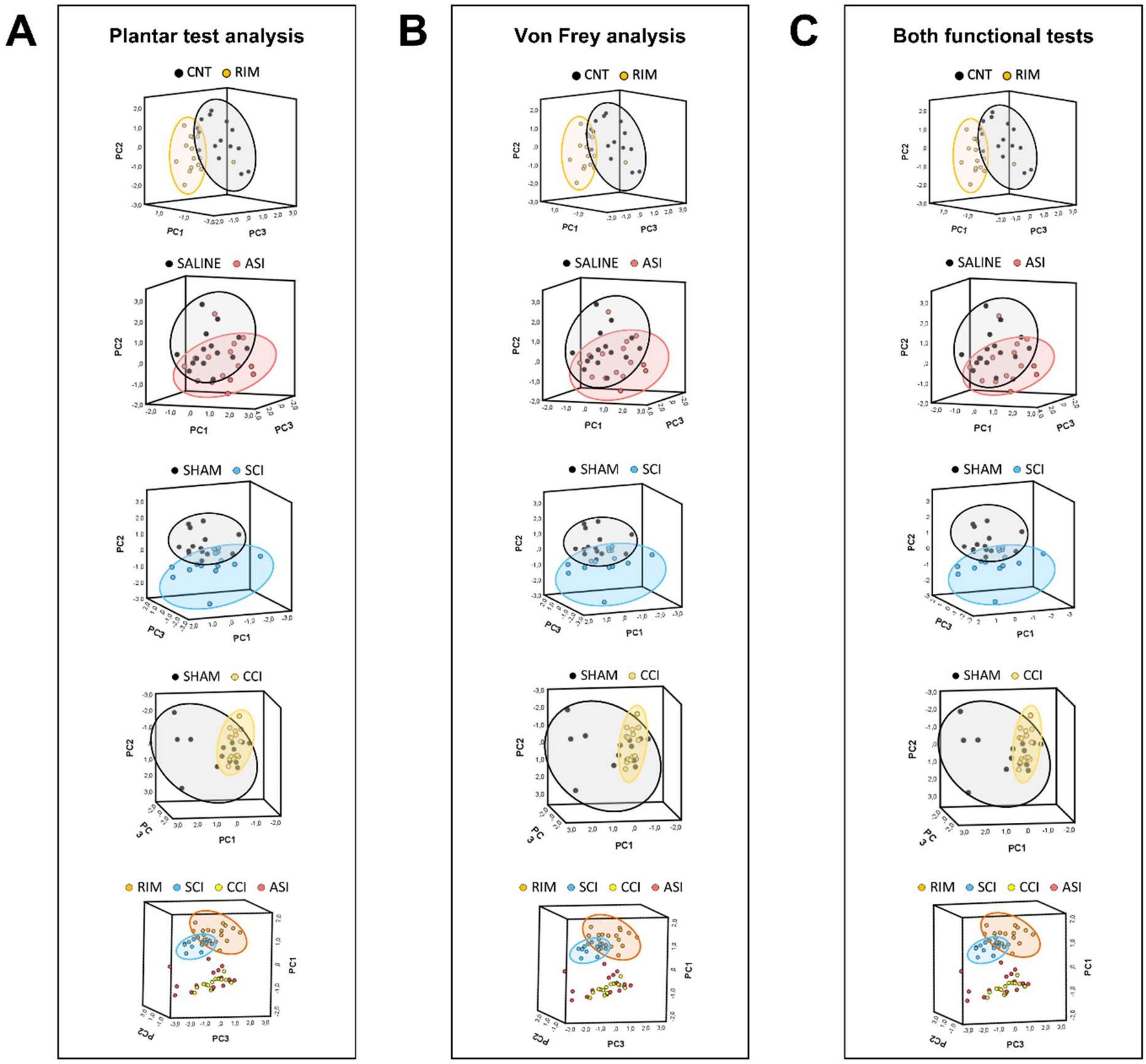

Fig. S3 shows the Intensities of the most relevant peaks found in mass spectra from injured mice serum samples obtained with the MALDI-7090 TOF. Data shown as the median of each group ± IQR. A significant difference was found in only one peak. a-c: groups not sharing a letter in the same peak are significantly different, $p<0.05$, by Duncan's or Kruskal-Wallis test.

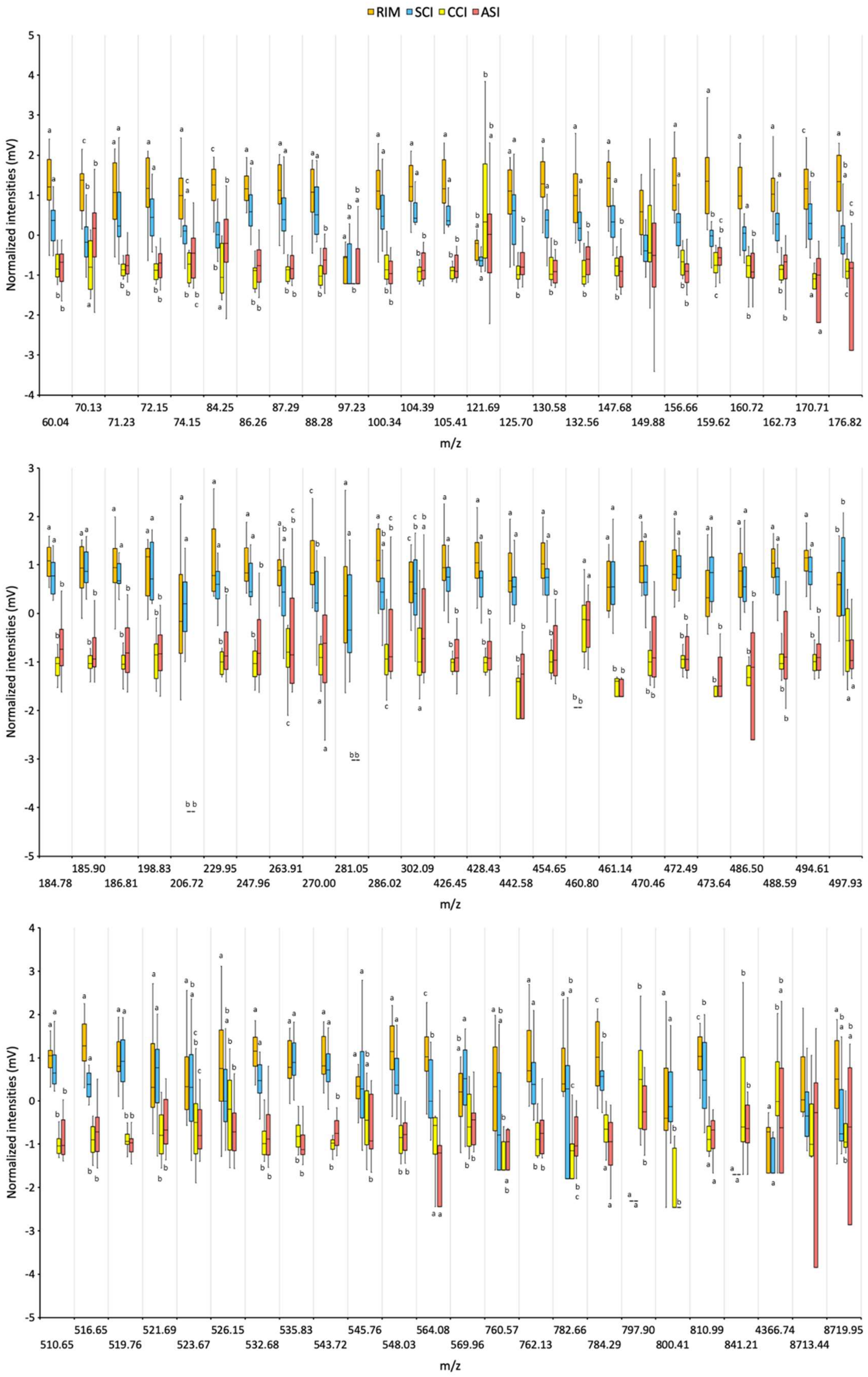

Supplement: Supplementary file 1 — cn2c00665_si_001.pdf [file cn2c00665_si_001.pdf]
